# Bmi-1 regulates mucin levels and mucin O-glycosylation in the submandibular gland of mice

**DOI:** 10.1371/journal.pone.0245607

**Published:** 2021-01-19

**Authors:** Akihiko Kameyama, Risa Nishijima, Kimi Yamakoshi

**Affiliations:** 1 Cellular and Molecular Biotechnology Research Institute, National Institute of Advanced Industrial Science and Technology (AIST), Tsukuba, Ibaraki, Japan; 2 Department of Mechanism of Aging, Research Institute, National Center for Geriatrics and Gerontology, Obu, Aichi, Japan; Cairo University, EGYPT

## Abstract

Mucins, the major components of salivary mucus, are large glycoproteins abundantly modified with O-glycans. Mucins present on the surface of oral tissues contribute greatly to the maintenance of oral hygiene by selectively adhering to the surfaces of microbes via mucin O-glycans. However, due to the complex physicochemical properties of mucins, there have been relatively few detailed analyses of the mechanisms controlling the expression of mucin genes and the glycosyltransferase genes involved in glycosylation. Analysis performed using supported molecular matrix electrophoresis, a methodology developed for mucin analysis, and knockout mice without the polycomb group protein Bmi-1 revealed that Bmi-1 regulates mucin levels in the submandibular gland by suppressing the expression of the mucin *Smgc* gene, and that Bmi-1 also regulates mucin O-glycosylation via suppression of the glycosyltransferase *Gcnt3* gene in the submandibular gland.

## Introduction

Mucins are large glycoproteins with a core protein modified with O-glycans, accounting for 50%–80% of the molecular mass, that are produced by the salivary glands and bind to adhesion molecules on the surfaces of microorganisms through mucin-type *O*-glycans and contribute to the maintenance of oral hygiene by selectively controlling the adhesion and colonization of microbes on the surface of oral tissues [1, 2]. However, in addition to a high molecular weight, mucins are difficult to handle and analyze because of the presence of highly dense glycans linked to markedly heterogeneous and branched structures. Therefore, to date, there have been relatively few detailed analyses of the mucins or the mechanisms controlling the expression patterns of mucin genes and glycosyltransferase genes involved in glycosylation.

The polycomb group protein Bmi-1, a component of polycomb-repressive complex 1 (PRC1), is involved in the self-renewal of certain types of adult stem cells by silencing gene loci [3–7]. A previous study by our group of *Bmi-1*-deficient knockout mice [8] found that the abundance of acinar cells is decreased due to reduced self-renewal ability of stem cells in the submandibular gland (SMG), which results in lower production and secretion of saliva [9]. However, the saliva produced by *Bmi-1*-knockout mice was significantly more viscous. Negatively charged glycans such as sialic acid allow a large amount of water molecules to be retained in the mucin molecule, which influences the polymerization of mucins and the ability to bind to various microorganisms [10, 11], resulting in increased viscosity of saliva and poorer oral hygiene. Therefore, Bmi-1 is a strong candidate to control expression of the mucin gene and the glycosyltransferase genes that transfer glycans to mucin molecules. Hence, elucidation of the mechanisms regulating the expression of these genes is likely to facilitate the development of new strategies for the prevention and intervention of various oral diseases, including xerostomia.

In the present study, supported molecular matrix electrophoresis (SMME), a method developed to separate high molecular weight glycoproteins [12], was used to elucidate the Bmi-1-associated mechanism underlying the regulation of the expression of the mucin and glycosyltransferase genes. Unlike polyacrylamide gel electrophoresis and agarose gel electrophoresis, SMME is useful to separate mucin molecules regardless of the molecular weights. Using this approach and knockout mice lacking expression of Bmi-1 protein revealed that Bmi-1 regulates mucin levels by suppressing the expression of mucin submandibular gland protein C (*Smgc*), a splicing variant of *mucin 19* in the SMG and that Bmi-1 also regulates mucin O-glycosylation via suppression of mucin-type glucosaminyl (N-acetyl) transferase 3 (*Gcnt3*) in the SMG.

## Methods

### Study approval

All animal studies were approved by Animal Care and Ethics Committee of the National Center for Geriatrics and Gerontology and adhered to the institutional animal protocol guidelines (approval no. 30–32, 31–29, 2–20).

### Mice

Bmi1^+/-^ (C57BL/6) mice were crossed to obtain WT and Bmi-1^-/-^ mice [8]. Male WT and Bmi-1^-/-^ mice were used at 12 weeks of age for histological and immunohistochemical analyses, at 4–9 weeks of age for SMME, mucin quantification, and glycan analysis, at 4–6 weeks of age for qRT-PCR analysis of mRNA expression levels, at 7–11 weeks of age for neuraminidase activity assays, and at 5–10 weeks of age for ChIP analysis. In addition, 3-day old WT mice were used for anti-Smgc antibody validation via immunohistochemical analysis. All mice were housed in a pathogen-free environment throughout the study period.

### Histological and immunohistochemical analyses

At the age of 12 weeks, the WT and Bmi-1^-/-^ (C57BL/6) mice [8] were sacrificed and the SMGs were immediately isolated, rinsed with phosphate-buffered saline (PBS), fixed in 4% paraformaldehyde (09154–85; Nacalai Tesque, Kyoto, Japan), and embedded in paraffin. The paraffin-embedded SMG tissues were cut into 4 μm-thick sections, which were then deparaffinized, rehydrated, and stained with hematoxylin and eosin (8659; Sakura Finetek Japan Co. Ltd., Tokyo, Japan) or the polyvalent basic dye Alcian blue (AB) (pH 1.0 [4086–2; Muto Pure Chemicals Co. Ltd., Tokyo, Japan] or 2.5 [4085–2; Muto Pure Chemicals Co. Ltd.]) or used for immunofluorescence analysis. For immunofluorescence analysis, the tissue sections were boiled for 10 min in 1 mM ethylenediaminetetraacetic acid (EDTA, 311–90075; Nippon Gene Co. Ltd., Tokyo, Japan) for antigen retrieval. After blocking for 30 min with Image-IT FX signal enhancer reagent (136933; Thermo Fisher Scientific, Waltham, MA, USA), the tissue sections were washed with ultrapure water and incubated overnight at 4°C with anti-Smgc antibody (PAB27571; Abnova Corporation, Taipei, Taiwan). Afterward, the tissue sections were washed with PBS and incubated with donkey anti-goat antibody against immunoglobulin G (Alexa Fluor 555; Thermo Fisher Scientific), while the nuclei were stained with the fluorescent stain 4’,6-diamidino-2-phenylindole (Dojindo Laboratories Co. Ltd., Kumamoto, Japan). The SMG and the pancreas of 3-day old WT mice were used for anti-Smgc antibody validation. Images were acquired using a BIOREVO BZ-9000 microscope (Keyence Corporation, Osaka, Japan) equipped with a CFI Plan Apo λ 40× objective lens (972033; Nikon Corporation, Tokyo, Japan) and BZ-II software (Keyence Corporation). Quantitative evaluation of the fluorescence area was performed by using the images without including granular convoluted ducts that were stained non-specifically positive. Fluorescence area above a threshold of brightness was quantitatively assessed using the BZ-X analyzer software package (Keyence Corporation).

### Enrichment of mucins from SMG

The SMGs were dissected from six individual WT and Bmi-1^-/-^ mice. Mucins were enriched from the SMG samples according to the procedure described in our previous report [13]. Briefly, acetone powders prepared from the SMG samples (approximately 50 mg) were suspended in PBS (314–90185; Nippon Gene Co. Ltd.) (pH 7.4, 500 μL) and centrifuged at 12,000 ×*g* for 10 min at 4°C. The supernatants were transferred into new tubes and mixed with a saturated aqueous solution of calcium acetate (402850; Sigma-Aldrich Corporation, St. Louis, MO, USA) at a 5:1 ratio. Then, the solutions were mixed with three volumes of ethanol (057–00456; Fujifilm Wako Pure Chemical Corporation, Osaka, Japan) kept at −80°C for 1 h and centrifuged at 15,000 ×*g* for 10 min at 4°C. The supernatants were discarded and the precipitates were suspended in 2 M urea (217–00615; Fujifilm Wako Pure Chemical Corporation) in PBS (pH 7.4, 100 μL) and centrifuged at 15,000 ×*g* for 10 min at 4°C. The supernatants were transferred into new tubes and ethanol precipitation was repeated as described above. The resulting precipitates were dissolved in 50 μL of 8 M urea in PBS (pH 7.4) and stored at 4°C until use.

### SMME

SMME membranes were prepared as described in a previous report [12]. Briefly, polyvinylidene difluoride membranes (Immobilon-P; pore size, 0.45 μm; EMD Millipore Corporation, Billerica, MA, USA) were cut in a rectangular shape (6 × 5 cm) and immersed in 0.1% hydrophilic polymer solution with gentle shaking for 2 h. The hydrophilic polymers used for glycan analysis and immunostaining were polyvinyl alcohol (PVA: average molecular weight, 22,000; Wako Pure Chemical Industries Ltd., Osaka, Japan) and a 1:3 mixture of PVA and polyvinylpyrrolidone (PVP: average molecular weight, 40,000; Sigma-Aldrich Corporation), respectively. The prepared SMME membranes were equilibrated using a running buffer of 0.1 M pyridine-formic acid (pyridine: 116–05316; Fujifilm Wako Pure Chemical Corporation; formic acid: 066–00461; Fujifilm Wako Pure Chemical Corporation) (pH 4.0) for 30 min with gentle shaking. Subsequently, the SMME membranes (separation length, 6 cm) were placed in a membrane electrophoresis chamber (EPC105AA; Advantec Toyo Kaisha Ltd., Tokyo, Japan), streaked with 1 μL of mucin enrichment solution (5 mm wide and 1.2 cm from the bottom of the membrane) and subjected to a constant current at 1.0 mA/cm for 30 min. For AB staining, immediately after electrophoresis, the membranes were immersed in 30% acetic acid (017–00256; Fujifilm Wako Pure Chemical Corporation) in methanol (134–14523; Fujifilm Wako Pure Chemical Corporation) for 30 min and subsequently incubated in AB dye solution (KT003; Cosmo Bio Co. Ltd., Tokyo, Japan) (pH 4.0) with gentle shaking for 30 min. The stained membranes were washed with methanol for a few minutes to remove the background color.

### Mucin quantification

The concentrations of mucin in the enrichment solutions were determined as N-acetylgalactosamine (GalNAc) concentrations [14]. The enrichment solutions were diluted by 5-fold with PBS and the mucin concentrations of the diluted solutions were determined with the use of a Fecal Mucin Assay Kit (Cosmo Bio Co. Ltd.) in accordance with the manufacturer’s instructions but without dextranase digestion. The intensities of fluorescence at 383 nm following excitation at 336 nm by reacting 2-cyanoacetamide with GalNAc were measured using a SpectraMax M5 plate reader (Molecular Devices LLC., San Jose, CA, USA). GalNAc concentrations were calculated from a standard curve prepared from authentic GalNAc solutions.

### Real-time quantitative polymerase chain reaction (qRT-PCR) analysis of mRNA expression levels

Total RNA was isolated from mouse SMG using TRI Reagent (TR118; Molecular Research Center Inc., Cincinnati, OH, USA). Contaminating genomic DNA was removed with the use of a RNeasy Mini Kit (74104; Qiagen, Hilden, Germany), and RNA was converted to complementary DNA (cDNA) using High-Capacity cDNA Reverse Transcription Kits (4374967; Thermo Fisher Scientific). The mRNA level was determined with the following primer probes of TaqMan™ Gene Expression Assays (Thermo Fisher Scientific) using a CFX96 Touch™ Real-Time PCR Detection System (Bio-Rad Laboratories, Hercules, CA, USA): *gapdh* (Mm99999915_g1); *Mucin1* (Mm00449604_m1); *Mucin2* (Mm01276696_m1); *Mucin3* (Mm01207064_m1); *Mucin4* (Mm00466886_m1); *Mucin5AC* (Mm01276718_m1); *Mucin5B* (Mm00466391_m1); *Mucin6* (Mm00725165_m1); *Ovgp1/Mucin9* (Mm00801489_m1); *Prol1/Mucin10* (Mm00500594_m1); *Mucin13* (Mm00495397_m1); *Emcn/Mucin14* (Mm00497495_m1); *Mucin15* (Mm00662032_m1); *Mucin16* (Mm Mm01177118_m1); *Mcam/Mucin18* (Mm00522397_m1); *Mucin19* (Mm01306462_m1); *Smgc* (splice variant of *Mucin19*) [15, 16] (Mm01303807_m1); *Mucin20* (Mm00524818_m1); *Gm9573/Mucin21* (Mm04279594_m1); *β3gnt3* (Mm00472247_g1); *Gcnt2* (Mm01324836_m1); *Gcnt3* (Mm00511233_m1); *ST3Gal I* (Mm00501493_m1); *ST3Gal II* (Mm00486123_m1); *ST6GalNAc I* (Mm01252949_m1); and *ST6GalNAc II* (Mm00486130_m1). A comparative quantitative algorithm was used to analyze the data. The Ct of the gene of interest in the WT or Bmi-1^-/-^ sample was compared relative to the Ct of *gapdh*.

### Immunoblotting of SMME membranes

Electrophoresed mucins were immobilized on the SMME membranes as described previously [17]. In brief, immediately after electrophoresis, the membranes were transferred into acetone and gently shaken for 30 min and then subjected to heat treatment (150°C for 5 min) using a TLC Thermal Blotter (ATTO Corp., Tokyo, Japan). Afterward, the membranes were blocked with 5% bovine serum albumin (A3059; Sigma-Aldrich Corporation), washed with PBS and Tween® (167–11515; Fujifilm Wako Pure Chemical Corporation) detergent (PBS-T), and then incubated overnight with antibodies at 4°C. After washing with PBS-T, the membranes were incubated with horseradish peroxidase-conjugated donkey anti-goat IgG antibody (705-035-147; Jackson Immuno Research Laboratories Inc., West Grove, PA, USA), washed, and visualized using chemiluminescence reagents (WP20005; Thermo Fisher Scientific or RPN2232; GE Healthcare, Chicago, IL, USA). The following primary antibodies were used: Muc 1 (sc-6826; Santa Cruz Biotechnology Inc., Dallas, TX, USA); Prol1/Muc 10 (ab119999; Abcam, Cambridge, UK); Muc 19 (ab121014; Abcam); and Smgc (PAB27571; Abnova Corporation).

### Glycan release and permethylation

AB-stained mucin bands were excised and subjected to reductive β-elimination, as described previously [12, 13]. Briefly, the excised bands were incubated in 40 μL of 500 mM NaBH_4_ (191–11452; Fujifilm Wako Pure Chemical Corporation) in 50 mM NaOH (197–14891; Fujifilm Wako Pure Chemical Corporation) at 45°C for 16 h and the reaction was subsequently quenched by the addition of 8 μL of glacial acetic acid (017–00256; Fujifilm Wako Pure Chemical Corporation). The solutions were desalinated using a cation-exchange solid phase extraction cartridge (Oasis MCX, 60 mg; Waters Corporation, Milford, MA, USA) and concentrated and co-evaporated using 1% acetic acid in methanol to remove boric acid. The resulting residues were permethylated using iodomethane (137–02663; Fujifilm Wako Pure Chemical Corporation) and NaOH, desalinated, and dried as previously reported [12, 13].

### Mass spectrometry (MS)

MS spectra were acquired using a matrix-assisted laser desorption/ionization time-of-flight (MALDI-TOF) mass spectrometer (UltraFlex; Bruker Daltonik GmbH, Bremen, Germany). Ions were generated by a pulsed 337-nm nitrogen laser and accelerated to 20 kV. All spectra were obtained in the positive reflectron mode and analyzed using FlexAnalysis 2.0 software (Bruker Daltonik GmbH). Glycan signals were assigned using the GlycoMod tool (https://web.expasy.org/glycomod/). Tandem mass spectra were acquired using a MALDI-quadrupole ion trap (QIT)-TOF mass spectrometer (AXIMA-QIT; Shimadzu Corp., Kyoto, Japan) in the positive ion mode. Ions were generated using a 337-nm nitrogen laser and argon gas was used as the collision gas for collision-induced dissociation. For sample preparation, 0.5 μL of 2,5-dihydroxybenzoic acid (DHB, 044–29101; Fujifilm Wako Pure Chemical Corporation) solution (10 mg/mL in 30% ethanol) was spotted onto a MALDI target plate and dried. The permethylated glycans were dissolved in 50% acetonitrile, and 0.5-μL aliquots of the solution were spotted onto the dried DHB crystal on the target plate and air dried. Tandem mass spectra of the permethylated glycans were assigned using the GlycoWorkbench 2.1 suite of software tools (the European Carbohydrates Database [EUROCarbDB] project: http://www.eurocarbdb.org/).

### Sialic acid linkage–specific alkylamidation (SALSA)

Mucin bands stained with AB were excised and subjected to reductive oximation as described previously [18]. The released *O*-glycan oximes were captured on hydrazide beads (BlotGlyco, 5 mg; Sumitomo Bakelite Co., Ltd., Tokyo, Japan) using the procedure recommended by the manufacturer. The sialic acid residues of the *O*-glycans on the beads were then differentially amidated with methylamine (132–01851; Fujifilm Wako Pure Chemical Corporation) for α2,3-sialic acids and isopropylamine (163–04863; Fujifilm Wako Pure Chemical Corporation) for α2,6-sialic acids using the SALSA method [19]. The derivatized *O*-glycans were liberated, labeled with aoWR (a component of BlotGlyco, Sumitomo Bakelite Co. Ltd.), and purified according to the manufacturer's instructions. The labeled *O*-glycans were analyzed by MALDI-TOF MS as described above.

### Neuraminidase activity

The neuraminidase activity of the mice SMG homogenates was determined using the EnzyChrom™ Neuraminidase Assay Kit (ENEU-100; BioAssay Systems, Hayward, CA, USA) according to the manufacturer’s instructions. The homogenates of the mice SMGs were prepared by homogenization in tissue lysis buffer consisting of 1% sodium dodecyl sulfate (28-3260-5; Sigma-Aldrich Corporation), 4 M urea (217–00615; Wako Pure Chemical Industries, Ltd.), 1 mM EDTA, 150 mM Tris (pH 8.0) (314–90065; Nippon Gene Co. Ltd.), and a protease inhibitor cocktail (25955; Nacalai Tesque, Inc.). Following sonication, the samples were centrifuged at 10,000 ×*g* for 15 min at 4°C, and the supernatant concentrations were measured. For the neuraminidase assays, 80 μL of working reagent, consisting of assay buffer, substrate, cofactors, enzyme, and dye reagent, were added to 20 μL of each sample (200 μg of protein) or various concentrations of sialic acid (400, 240, 120, and 0 μM). Appropriate controls (blank, H_2_O) were also included in the assay. The reaction mixture was incubated at 37°C. After 20 min, the optical density (OD_20 min_) was measured at 570 nm. The reaction mixture was incubated for an additional 30 min at 37°C, and OD was measured again (OD_50 min_) at 570 nm. The OD of the sample, blank, and H_2_O control at 20 min were subtracted from the OD of the sample, blank, and H_2_O control at 50 min to obtain the ΔR_sample_, ΔR_blank_, and ΔR_H2O_ values, respectively. The OD of the standard was plotted against the standard concentration, and the neuraminidase activity of the sample was calculated using the following equation:

Neuraminidase activity (units/L) = (ΔR_SAMPLE_ − ΔR_BLANK_ − ΔR_H2O_)/Slope × 1/t, where slope refers to the slope of the standard curve in μM^−1^ and *t* is the reaction time between readings (30 min).

### Chromatin immunoprecipitation (ChIP) analysis

The mouse SMGs were minced in microcentrifuge tubes, washed with 1 ml of PBS, and centrifuged at 500 x *g* for 5 min at 4°C. The supernatants were discarded and the nuclei of the SMG cells were extracted from the precipitates with the use of NE-PER Nuclear and Cytoplasmic Extraction Reagents (78835; Thermo Fisher Scientific) in accordance with the manual supplied by the manufacturer with some modifications. Briefly, the precipitates were homogenized using a tissue grinder in an appropriate volume of cytoplasmic extraction reagent (CER)-Ⅰ, vortexed for 15 s and incubated on ice for 10 min. Then, CER-Ⅱ was added to the tubes, which were vortexed for 5 s and then incubated on ice for 1 min. The sample solutions were vortexed again for 5 s, passed through 100-μm and 40-μm filters (352360 and 352340; Corning Incorporated, Corning, NY, USA) in order and centrifuged at 1300 × *g* for 5 min at 4°C. Then, the supernatants were discarded and the precipitates were used in the next step to obtain sheared chromatin as described in the manual included with the truChIP Chromatin Shearing Reagent Kit (520154; Covaris, Inc., Woburn, MA, USA) but with some modifications. Briefly, the precipitates were suspended in Fixing Buffer A with freshly prepared 11.1% formaldehyde solution (final concentration of 1%), and crosslinked at room temperature for 5min. Subsequently, following the addition of Quenching Buffer E to quench the crosslinking reaction, the tubes were kept at room temperature for an additional 5 min and were then centrifuged at 1700 ×*g* for 5 min at 4°C. Afterward, the supernatants were discarded and the precipitates were rinsed with Shearing Buffer D3 containing protease inhibitors, then centrifuged again at 1700 ×*g* for 5 min at 4°C. The supernatants were discarded again and the precipitates were suspended in Shearing Buffer D3, then transferred to adaptive focused acoustics tubes (520045; Covaris Inc.). The chromatin was sheared with a Covaris M220 Focused-ultrasonicator (Covaris Inc.), centrifuged at 12,000 x *g* for 15 min at 4°C, and the supernatants were transferred into pre-chilled new tubes. The chromatin was incubated with a mouse antibody against Bmi-1 (ab14389; Abcam) or a mouse IgG antibody (#5415; Cell Signaling Technology, Danvers, MA, USA) at 4°C overnight. The next day, immune complexes were bound to pre-blocked Protein G Mag Sepharose® magnetic beads (28951379; GE Healthcare) at 4°C for 2 h. Afterward, the precipitates were thoroughly washed, suspended in elution buffer (50 mM Tris [pH 8.0], 10 mM EDTA, 1% sodium dodecyl sulfate, 200 mM Sodium Chloride) and extracted by incubation at 65°C for 15 min. To remove crosslinks, the DNA–protein complexes were transferred into new tubes, incubated at 65°C for 6 h followed by incubation with 0.05 mg mL^-1^ RNase A (R6513; Sigma-Aldrich Corporation) at 37°C for 20 min and with 0.1 mg mL^-1^ Proteinase K (160–22752; Wako Pure Chemical Industries) at 55°C for 30 min. DNA was purified using the MinElute PCR Purification Kit (28004; Qiagen) and quantified by qRT-PCR analysis. The ratio of antibody-precipitated chromatin DNA to the total DNA amount (% input) was determined with TaqMan minor groove binder (MGB) probes and primers (Thermo Fisher Scientific) using a CFX Connect™ Real-Time PCR Detection System (Bio-Rad Laboratories). The following TaqMan MGB probes with the actual sequences were used:

*Smgc* locus
Set 1 (intron 1): 5’-CAGTTTCTGAAGGTCTC-3’
Set 2 (intron 13): 5’-AGTGCTCTTAATAACCC-3’
*Gcnt3* locus
Set 1 (promoter): 5’-TATGACGCCAATCAC -3’
Set 2 (exon 3): 5’-CCCTCAGACTGAAGTGTGA -3’
PCR primer sequences used are as follows:
*Smgc* locus
Primer set 1: 5’-TCAACTTGAGCACGCATCTTG-3’(forward), 5’-CATTGCCCTTCCACCATACAG-3’(reverse)
Primer set 2: 5’-CTGACGAATTGGACCATGTGATT-3’(forward), 5’-TCCTGGTGTGATTAGTGGGTACAA-3’(reverse)
*Gcnt3* locus
Primer set 1: 5’-CCCTTACCTCTGGCAGAAGGA-3’(forward), 5’-GGTATGCAGCCTCGGGTCTA-3’(reverse)
Primer set 2: 5’-GCCATCCTTGCCCTGAAAC-3’(forward), 5’-CCAGATCCATGGCATCGAA-3’(reverse)

### Statistical analysis

Statistical significance of mucin levels and neuraminidase activity was determined by a student’s *t*-test; *P*-values < 0.05 were considered significant. Statistical significance of qRT-PCR analysis, glycan analysis, and ChIP analysis was determined using a student’s *t*-test with Bonferroni test for post hoc comparisons when significance was determined by analysis of variance. The numbers of samples analyzed (*n*) in each experiment are listed in the Figure Legends. Statistical tests were performed using GraphPad Prism software (GraphPad Software Inc., San Diego, CA, USA).

## Results

### SMG acinar cells of Bmi-1^-/-^ mice were strongly stained by Alcian Blue at pH 2.5, as compared with those of WT mice

**Fig 1:**
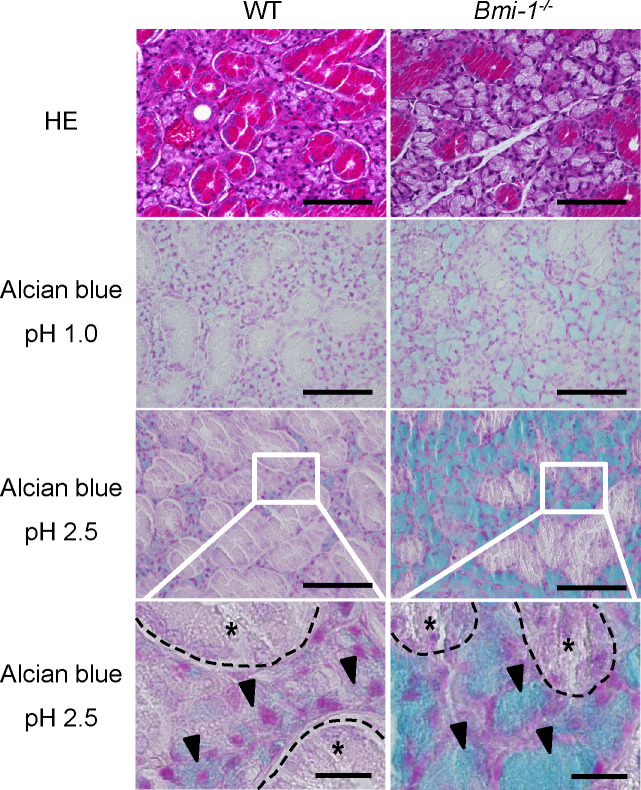
SMG acinar cells of Bmi-1^-/-^ mice were more strongly stained with AB at pH 2.5, as compared with those of WT mice. Hematoxylin and eosin (top panels), AB at pH 1.0 (upper middle panels), and AB at pH 2.5 (lower middle panels) staining analysis of SMG sections from WT and Bmi-1^-/-^ mice (12 weeks). Arrowheads indicate acinar cells. The structures of granular convoluted ducts are marked by asterisks and dashed lines. Scale bars, 100 μm (top, upper middle, lower middle panels) and 20 μm (magnified views in bottom panels).

The SMGs of the WT and Bmi-1^-/-^ mice were stained with hematoxylin and eosin. The cytoplasm of the SMG acinar cells of the Bmi-1^-/-^ mice was more lightly stained with eosin than that of the WT mice (Fig 1, Top). Eosin binds to positively charged biological substances, but not to mucus polysaccharides, such as mucins and glycosaminoglycans. These results suggest that SMG acinar cells of Bmi-1^-/-^ mice contain very few positively charged proteins and/or many mucus polysaccharides. Therefore, the SMG tissues of WT and Bmi-1^-/-^ mice were stained with AB dye, which binds to the carboxy and sulfate groups of acidic mucus polysaccharides. AB dye only binds to the sulfate groups at pH 1.0 and to the carboxy and sulfate groups at pH 2.5. Mucin molecules often have sialic acid moieties that contain carboxy groups at the ends of the glycans. On the other hand, glycosaminoglycans are classified as hyaluronic acid with only carboxy group or as proteoglycans with many carboxy and sulfate groups. That is, since mucin and hyaluronic acid are positively stained by AB at pH 2.5 and proteoglycans are positively stained by AB at pH 1.0 and 2.5, it is possible to roughly estimate the abundance of mucins, hyaluronic acid, and proteoglycans by staining with AB. At pH 1.0, SMG acinar cells of Bmi-1^-/-^ mice were stained slightly more strongly than those of WT mice, although there was no significant difference in staining intensity (Fig 1, upper middle). However, at pH 2.5, the SMG acinar cells of Bmi-1^-/-^ mice were stained much more strongly than those of WT mice (Fig 1, lower middle), suggesting that the amount of mucin and/or of hyaluronic acid and/or the number of sialoglycans in mucin, not proteoglycans, were significantly increased in SMG acinar cells of Bmi-1^-/-^ mice when compared with those of WT mice.

### AB-positive mucus polysaccharide components of SMG tissues of Bmi-1^-/-^ mice

**Fig 2:**
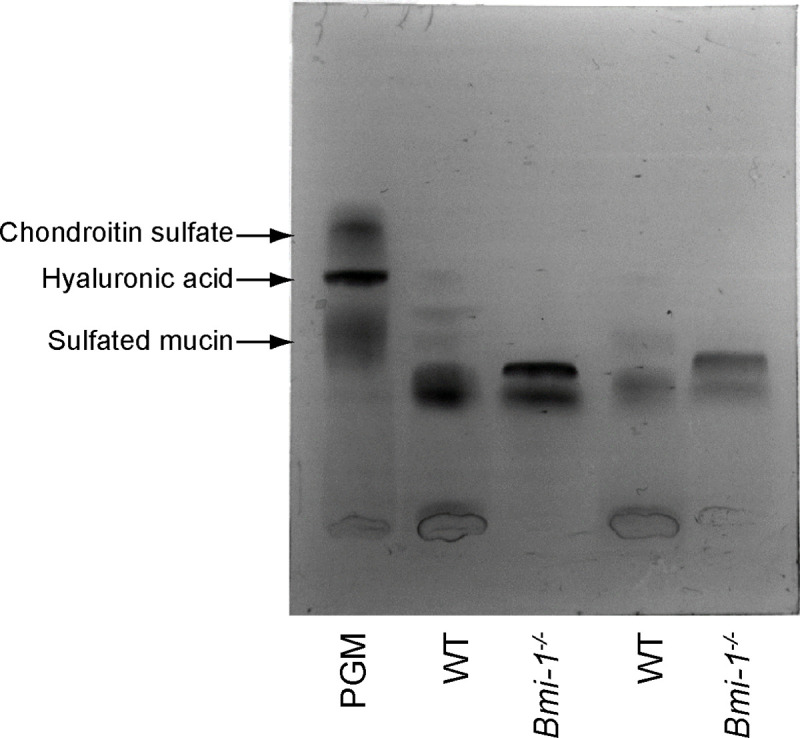
SMME analysis of SMG mucins of WT and Bmi-1^-/-^ mice. Comparison of SMG mucins of WT and Bmi-1^-/-^ mice (5 weeks) separated by SMME. All bands are from different individuals. Reference mucin: porcine gastric mucin (PGM). The SMME membrane was stained with AB (pH 4.0).

Given that the amount of mucin and/or hyaluronan and/or the number of sialoglycans in mucin were significantly increased in Bmi-1^-/-^ SMG acinar cells, a more biochemical method, SMME [12] was used to identify the components of acidic mucus polysaccharides that bind to AB dye. SMME allows the separation of mucus polysaccharides into proteoglycans, hyaluronic acid, sulfated mucins, and neutral mucins, which are all components of partially purified porcine gastric mucin (PGM), reported previously [12]. PGM was used as a reference sample in SMME, although neutral mucins cannot be stained with AB dye [20]. Consistent with the results of our previous studies [13, 21], bands of acidic mucins were detected in the acinar cells of both WT and Bmi-1^-/-^ mice (Fig 2 and S1A Fig). However, the bands of Bmi-1^-/-^ mice were stronger than those of WT mice (Fig 2 and S1A Fig). To confirm that these bands were indeed mucins, the bands were excised from the membranes and MALDI-TOF MS analysis of the released glycans was performed. *O*-glycans were detected in the bands of both WT and Bmi-1^-/-^ mice (S1B Fig and Table 1). Therefore, these bands were confirmed as mucins. These results indicate that mouse SMG acinar cells mainly contain mucins and SMGs of Bmi-1^-/-^ mice have a relatively increased abundance of mucins and/or glycans with acidic residues.

**Table 1:** Signals assigned to *O*-glycans in the MS of permethylated alditols obtained from excised bands.

Glycan No.	Obsd. (*m/z*)	Clcd.^a^ (*m/z*)	Glycan composition	Classification^b^
1	534.31	534.29	(Hex)_1_(HexNAc)_1_	N
2	895.56	895.46	(Hex)_1_(HexNAc)_1_(NeuAc)_1_	S
3	1140.69	1140.59	(Hex)_1_(HexNAc)_2_(NeuAc)_1_	S
4	1170.69	1170.60	(Hex)_1_(HexNAc)_2_(NeuGc)_1_	S
5	1256.72	1256.64	(Hex)_1_(HexNAc)_1_(NeuAc)_2_	S
6	1501.89	1501.77	(Hex)_1_(HexNAc)_2_(NeuAc)_2_	S

^a^ All mass to charge ratios (*m/z*) were calculated as [M + Na]^+^ ions of the corresponding permethylated alditols.

^b^ N: Neutral glycan; S: sialoglycan

Obsd: observed; Clcd: calculated

### Muc10 and Smgc expression levels are markedly increased in SMGs of Bmi-1^-/-^ mice

**Fig 3:**
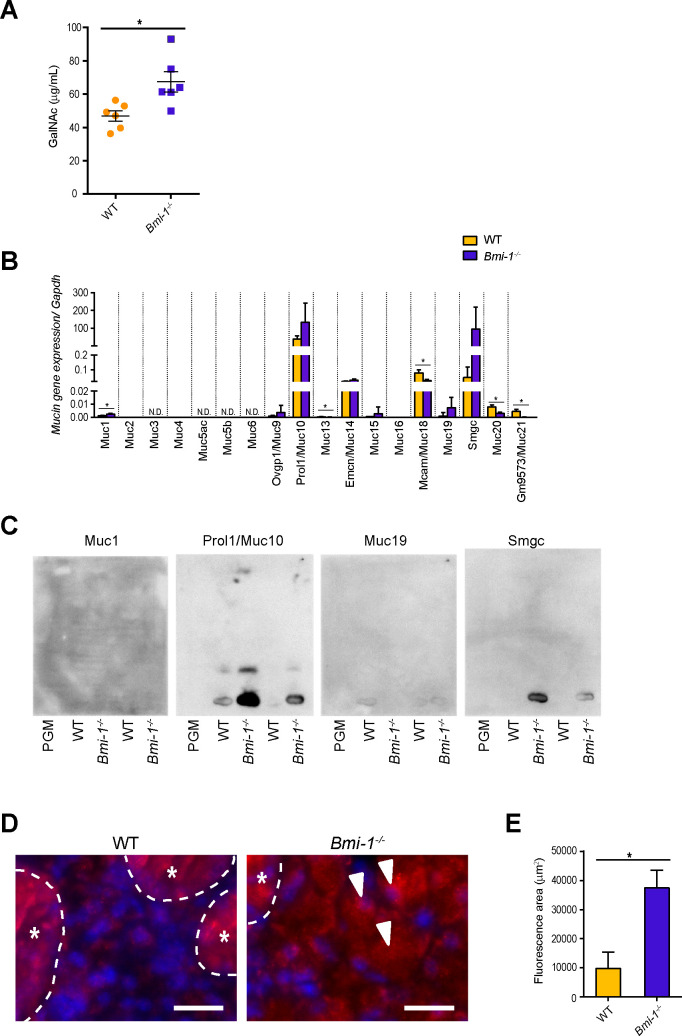
Increased mucin levels in the SMG tissues of Bmi-1^-/-^ mice. (A) Quantitative analysis of mucins in the SMG tissues of WT and Bmi-1^-/-^ mice. The bars represent the average amount of mucin in the SMG tissues of six individual WT and Bmi-1^-/-^ mice (Fig 2, *n* = 2 and S1A Fig, *n* = 4). Data are presented as mean ± standard error (SE). *P < 0.05. (B) The expression of mucin genes in the SMG of WT and Bmi-1^-/-^ mice. The mRNA levels of each mucin gene relative to *gapdh* in SMG of WT and Bmi-1^-/-^ mice (4–6 weeks). Data are presented as mean ± SE; *n* = 5 or 6. * *P* < 0.0028. N.D; not detected (C) Antibody staining of SMME membranes from SMG fractions of WT and Bmi-1^-/-^ mice with antibodies against Mucin 1, Muc 10/Prol 1, Muc 19, and Smgc. SMG samples and SMME experimental conditions are the same as in Fig 2. (D) Immunohistochemical analysis of Smgc (red) expression of SMGs from WT and Bmi-1^-/-^ mice (12 weeks). DNA was stained with 4’, 6-diamidino-2-phenylindole (blue). Arrowheads indicate positive acinar cells. Ductal structures are marked by dashed lines and asterisks. The granular convoluted ducts were stained non-specifically positive. Scale bars, 20 μm (E) Quantitative analysis of areas stained by immunohistochemistry in (D). Data represent means ± SE of three independent images for each SMG; *n* = 3. * *P* < 0.05.

To determine whether Bmi-1^-/-^ SMGs have increased mucin levels as compared to WT SMGs, the amounts of mucins in SMGs of WT and Bmi-1^-/-^ mice were quantified. The amount of mucin was greater in Bmi-1^-/-^ mice than WT mice (Fig 3A). There are approximately 20 genes that encode the core protein of mouse mucins [22, 23]. Due to the possibility of greater amount of mucins in the SMG tissues of Bmi-1^-/-^ mice, whether the amount of the mucin core protein increased in the SMG tissues of Bmi-1^-/-^ mice by reacting the SMME membranes with antibodies against mucins was determined. qRT-PCR was performed to determine the expression levels of 18 mucin genes in the SMG tissues of WT and Bmi-1^-/-^ mice. In the SMG tissues of the WT and Bmi-1^-/-^ mice, the expression levels of *Prol1/Muc10* was highest, while those of *Emcm/Muc14*, *Mcam/Muc18*, and *Smgc* (splice variant of *Muc19*) [15, 16, 24] were also relatively high (Fig 3B). However, the expression levels of *Muc2*, *Muc4*, and *Muc16* were very low, while *Muc3*, *Muc5ac*, *Muc5b*, and *Muc6* were not detected in the SMG tissues of WT and Bmi-1^-/-^ mice (Fig 3B). The expression levels of *Muc13*, *Mcam/Muc18*, *Muc20*, and *Gm9573/Muc21* were significantly lower in the SMG tissues of Bmi-1^-/-^ mice as compared to WT mice, whereas the expression levels were approximately 1.8-fold higher for *Muc1*, 3.5-fold for *Prol1/Muc10*, 6.4-fold for *Muc19*, and 1968-fold for *Smgc* in the SMG tissues of Bmi-1^-/-^ mice as compared to WT mice (Fig 3B). However, although the expression levels of *Prol1/Muc10*, *Muc19*, and *Smgc* were increased in the SMG tissues of Bmi-1^-/-^ mice, these differences were not statistically significant (Fig 3B). These findings suggest that Bmi-1 deficiency altered the expression levels of these mucin genes. Next, the SMME membranes were reacted with antibodies against Muc1, Prol1/Muc10, Muc19, and Smgc.

The antibody against Prol1/Muc10 specifically reacted with the bands detected in both WT and Bmi-1^-/-^ mice but more strongly with bands of the Bmi-1^-/-^ mice (Fig 3C and S2 Fig), whereas antibodies against Muc1, Muc19, and Smgc did not react with those bands (Fig 3C and S2 Fig). Although Smgc was not detected by immunoblotting of the SMME membranes, gene expression analysis showed that Smgc expression was markedly higher in the SMG tissues of Bmi-1^-/-^ than those of WT mice (Fig 3B). Immunofluorescence staining analysis revealed that Smgc was strongly expressed in the SMG acinar cells of Bmi-1^-/-^ mice (Fig 3D and 3E and S3 Fig). These results indicate that the expression levels of Prol1/Muc10 and Smgc are remarkably increased in SMG of Bmi-1^-/-^ mice, and the increased concentrations of these mucins at least partly account the increased viscosity of saliva of Bmi-1^-/-^ mice.

### The major glycan of SMG mucins in Bmi-1^-/-^ mice is a sialoglycan with a structure not found in WT mice

**Fig 4:**
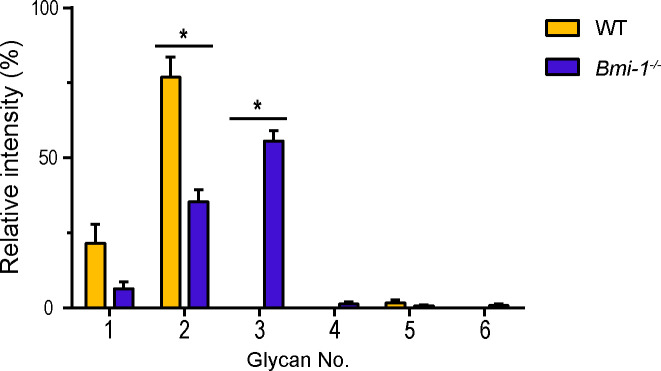
Glycan profiles of SMG mucins from WT and Bmi-1^-/-^ mice. Average glycan profiles in MALDI MS of SMG mucin bands of six individual WT and Bmi-1^-/-^ mice (Fig 2, *n* = 2 and S1A Fig, *n* = 4). Bars represent signal intensities of permethylated glycans. Data are presented as mean ± SE. Statistical significance was defined at * *P* < 0.0083 following Bonferroni correction.

Given the possible increase in glycans with acidic residues in the SMG tissues of Bmi-1^-/-^ mice, the glycan profiles of the SMG mucins of WT and Bmi-1^-/-^ mice were compared based on the signal intensities obtained by MALDI-TOF MS (S1B Fig and Table 1). The major glycan of SMG mucins of the WT mice was the sialoglycan (Hex)^1^(HexNAc)^1^(NeuAc)^1^ at *m/z* 895 (Fig 4 and Table 1). Interestingly, the main glycan of the SMG mucin of Bmi-1^-/-^ mice was the sialoglycan (Hex)^1^(HexNAc)^2^(NeuAc)^1^ at *m/z* 1140, which had a glycan structure not present in the WT mice (Fig 4 and Table 1). In addition, Bmi1^-/-^ mice had a higher proportion of sialoglycans than WT mice (Fig 4 and Table 1). The results suggest that the reason for the increased salivary viscosity and deep (pH 2.5) and weak (pH 1.0) staining of SMGs by AB in Bmi-1^-/-^ mice is that in addition to the increased mucin content, the abundance of sialic acid residues in the mucin is also increased.

### Structural analysis of the major glycan of the SMG mucin of Bmi-1^-/-^ mice

**Fig 5:**
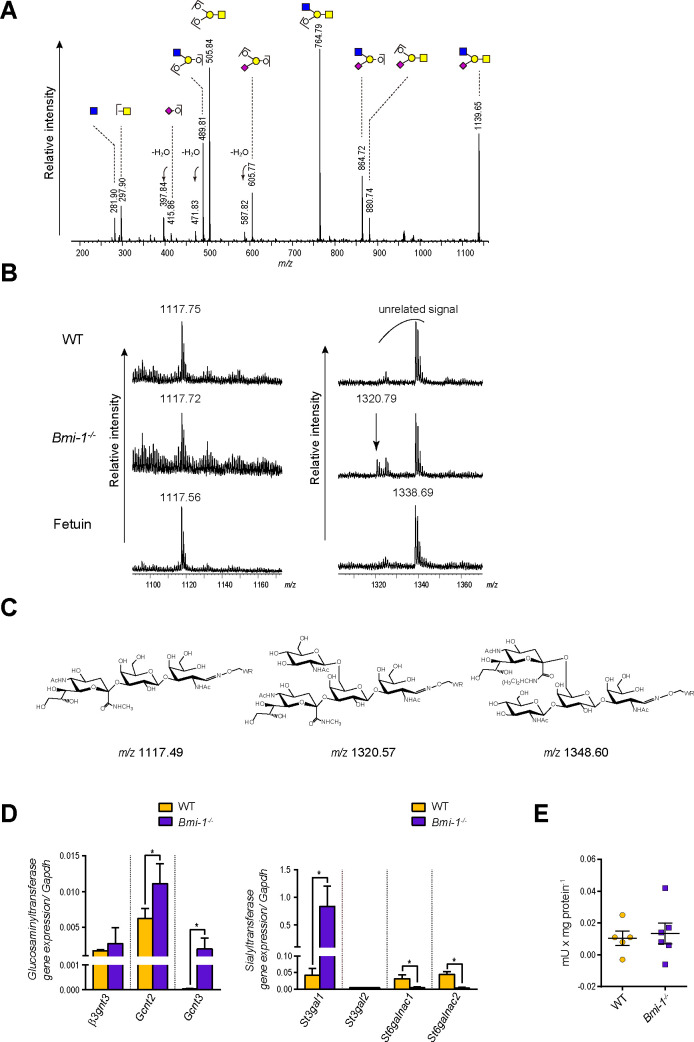
Structural analysis of mucin major glycan observed in SMG of Bmi-1^-/-^ mice. (A) MS/MS spectrum of the signal at *m/z* 1140. The signal 3 in S1B Fig was used as the precursor ion. Glycan structures are depicted using Consortium for Functional Glycomics (CFG) graphical notation for glycans. (http://www.functionalglycomics.org/static/consortium/CFGnomenclature.pdf). (B) Linkage analysis of sialic acids of the glycan by SALSA. Enlarged MS spectra of the modified sialoglycans are shown. The glycan from fetuin was used as a reference for [NeuAc(ɑ2–3)]Galβ1-3GalNAc. Left: (Hex)(HexNAc)(NeuAc), right: (Hex)(HexNAc)_2_(NeuAc). Indicated *m/z* values are observed values. The peak at *m/z* 1338.69 is an artifact signal. (C) The expected chemical structures and their predicted *m/z* values of the modified sialoglycans by SALSA. “WR” at the reducing end indicates tryptophanyl arginine. (D) The mRNA levels of each glucosaminyltransferase and sialyltransferase gene relative to *gapdh* in SMG tissues of WT and Bmi-1^-/-^ mice (4–6 weeks). Data are presented as mean ± SE; *n* = 5 or 6. Statistical significance was defined at *P < 0.0167 for glucosaminyltransferase and * *P* < 0.0125 for sialyltransferase following Bonferroni correction. (E) Quantification of neuraminidase activity in WT and Bmi-1^-/-^ mice (7–11 weeks) as mU per mg protein. Data are presented as mean ± SE; *n* = 5 or 6.

To elucidate the structure of the major glycan of the SMG mucin of Bmi-1^-/-^ mice, tandem MS of the signal at *m/z* 1140 was performed (Fig 5A). All the major fragments could be assigned and it is predicted that the structure of (Hex)_1_(HexNAc)_2_(NeuAc)_1_ at *m/z* 1140 is a core-1 type glycan, in which both of the NeuAc and HexNAc are bound to the Gal residue of core-1 structure “Galβ1-3GalNAc”. Further elucidation of the structure required a linkage analysis of the NeuAc by SALSA method in which sialic acids were differentially amidated with methylamine for α2-3-sialic acids and isopropylamine for α2-6-sialic acids [19, 25]. The method requires intact O-glycans with reducing ends to immobilize the O-glycans onto the polymer beads. The intact O-glycans, however are difficult to obtain from glycoproteins without involving glycan degradation referred to as peeling. Hydrazinolysis using anhydrous hydrazine has been used for obtaining the intact O-glycans, but it requires completely anhydrous conditions and careful handling owing to the toxic and hazardous properties of anhydrous hydrazine. Recently, a rapid and safe method to replace hydrazinolysis, the eliminative oximation method was reported [18]. In this study, immobilized O-glycans released by the eliminative oximation onto a polymer beads were modified using the SALSA method and analyzed by MALDI MS. MS spectra of the modified sialoglycans by SALSA showed that the signal at *m/z* 1320 corresponding to the methylamide derivative of the (Hex)_1_(HexNAc)_2_(NeuAc)_1_ was observed only in Bmi-1^-/-^ mice (Fig 5B). The expected chemical structures of the modified sialoglycans by SALSA were depicted in Fig 5C, respectively. The results suggest that the NeuAc is bound to the Gal residue with α2–3 linkage, indicating that the GlcNAc residue is probably bound to the 6-position of the same Gal residue of the glycan, considering the biosynthetic pathway of O-glycans. These findings are consistent with the results of gene expression analysis and neuraminidase activity in the SMG tissues of the WT and Bmi-1^-/-^ mice. The expression levels of *Gcnt2* gene encodes the I-branching enzyme, and *Gcnt3* gene encodes a member of the N-acetylglucosaminyltransferase family, both of which have β-1,6-N-acetylglucosaminyltransferase activity [26] (GlycoGene Database (GGDB), https://acgg.asia/ggdb2/), were about 1.8- and 65-fold greater, respectively in Bmi-1^-/-^ mice when compared with WT mice (Fig 5D). In addition, the expression level of β-galactoside α-2,3-sialyltransferase 1 (*ST3gal1*) gene [27] (GGDB, https://acgg.asia/ggdb2/) was about 20-fold greater in Bmi-1^-/-^ mice as compared to WT mice, while the expression levels of N-acetylgalactosaminide α-2,6-sialyltransferase 1 (*ST6galnac1*) and N-acetylgalactosaminide α-2,6-sialyltransferase 2 (*ST6galnac2*) gene [27] (GGDB, https://acgg.asia/ggdb2/) were significantly decreased in Bmi-1^-/-^ mice (Fig 5D). On the other hand, there was no significant difference in neuraminidase activity [28, 29] between the WT and Bmi-1^-/-^ mice (Fig 5E). Based on these results, it became clear that the structure of the main glycan of the SMG mucin of Bmi-1^-/-^ mice was GlcNAcβ1–6[NeuAc(α2–3)]Galβ1-3GalNAc, which is a glycan structure not present in the SMG mucin of WT mice.

### Bmi-1 suppresses the expression of *Smgc* gene and *Gcnt3* gene

**Fig 6:**
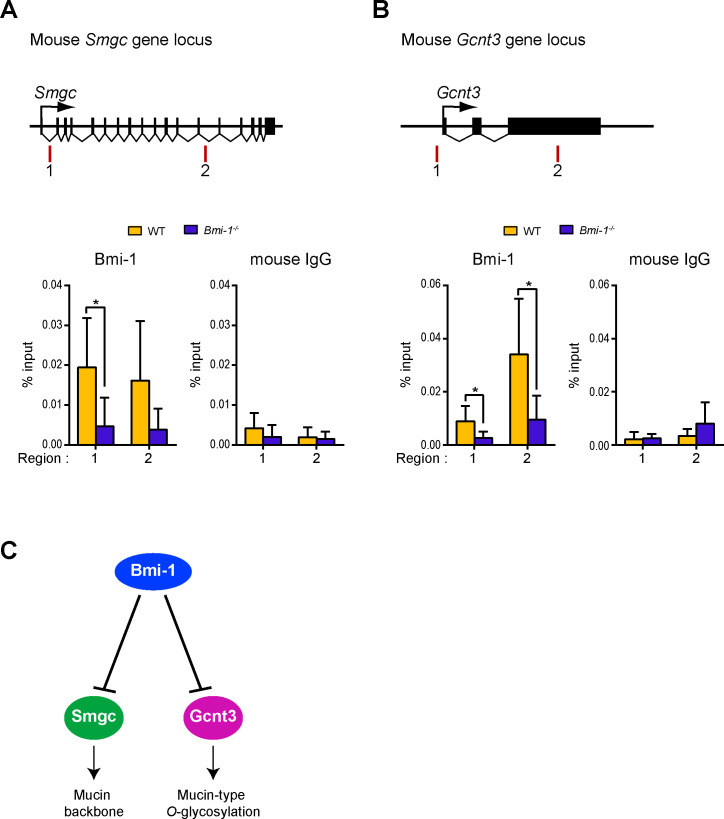
ChIP analysis using SMGs of WT and Bmi-1^-/-^ mice. (A) Schema of the mouse *Smgc* locus and ChIP analysis used to detect binding of Bmi-1 to the *Smgc* locus in the SMG of WT and Bmi-1^-/-^ mice. Amplified regions are shown as red bars. Data are presented as means ± SE; *n* = 4 or 6. * *P* < 0.025 (B) Schema of the mouse *Gcnt3* locus and ChIP analysis used to detect binding of Bmi-1 to the *Gcnt3* locus in the SMG of WT and Bmi-1^-/-^ mice. Amplified regions are shown as red bars. Data are presented as means ± SE; *n* = 4 or 6. * *P* < 0.025 (C) A model demonstrating how Bmi-1 may control mucin synthesis.

Bmi-1 is a component of PRC1 that silences a particular gene locus [30–32]. The expression levels of *Smgc* and *Gcnt3* were significantly higher in the SMG tissues of Bmi-1-/- mice as compared to those of WT mice (Figs 3B, 3D and 5D). These results led us to speculate that Bmi-1 normally represses the expression of these genes. To test this idea, the binding of Bmi-1 to the *Smgc* and *Gcnt3* loci of the SMG tissues of WT and Bmi-1^-/-^ mice was examined. The results showed that Bmi-1 binding to the *Smgc* and *Gcnt3* loci was substantially reduced in Bmi-1^-/-^ mice as compared to WT mice (Fig 6A and 6B), indicating that Bmi-1 normally suppresses expression of the *Smgc* and *Gcnt3* genes.

## Discussion

The PcG protein Bmi-1 is a component of PRC1 that is involved in the self-renewal of certain types of tissue stem cells via gene silencing [3–7]. The saliva of Bmi-1^-/-^ mice [8] was extremely viscous during the course of this study. Mucins, which are a major component of salivary mucus, are large glycoproteins with core protein modified with O-glycans that account for 50%–80% of the molecular mass [1]. Mucins bind to adhesion molecules on the surfaces of microorganisms through mucin-type O-glycan modifications and contribute to the maintenance of oral hygiene by selectively controlling the adhesion and colonization of microbes on the surface of oral tissues [1, 2]. Negatively charged glycans, such as sialic acid, promote retention of water molecules in the mucin molecules, which influences both the polymerization of mucins and their ability to bind to various microorganisms [10, 11], resulting in increased saliva viscosity and poor oral hygiene. Therefore, the increase in salivary viscosity observed in Bmi-1^-/-^ mice led us to speculate that Bmi-1 may be involved in the regulation of the expression of mucin or glycosyltransferase genes. However, due to the complex physicochemical properties of mucin, to date there have been relatively few detailed analyses of glycan profiles of mucins or the mechanisms controlling the expression of mucin genes and glycosyltransferase genes involved in glycosylation.

To circumvent this problem, in present study SMME was used, which allows electrophoresis of mucins regardless of molecular weights [12]. Using this methodology and knockout mice lacking Bmi-1 expression, it was revealed that Bmi-1 suppressed the expression of *Gcnt3*, which encodes a glycosyltransferase that can transfer GlcNAc to the 6-position of Gal; furthermore, high level of *Gcnt3* expression in the absence of Bmi-1 resulted in the formation of GlcNAcβ1–6[NeuAc(α2–3)]Galβ1-3GalNAc, a glycan structure not normally present in the SMG of WT mice (Fig 6C). Additionally, Bmi-1 was found to suppress the expression of *Smgc*, a splicing variant of *mucin 19*, in the SMG of mice and that *Smgc* was highly expressed in the absence of Bmi-1 (Fig 6C). SMG of Bmi-1^-/-^ mice demonstrated increased *Smgc*, *Muc10*, and sialoglycans levels, likely leading to the observed increase in salivary viscosity in Bmi-1^-/-^mice (Fig 6C). In addition, the results of our previous study found that salivary output was reduced in Bmi-1^-/-^ mice [9]. This suggested that the increased saliva viscosity and reduced amount of saliva observed in Bmi-1^-/-^ mice are similar to the symptoms of xerostomia. Xerostomia is defined as the subjective perception of dry mouth that is typically associated with increased salivary viscosity and reduced salivary flow [33–36], which cannot only causes oral disease and poor oral hygiene [37, 38] but also has systemic adverse effects, such as increased risk of aspiration pneumonia [39, 40]. Bmi-1^-/-^ mice have hematopoietic defects and neurological abnormalities, which results in death within 20 weeks after birth [8]. Interestingly, histological analysis of moribund Bmi-1^-/-^ mice revealed pneumonia, anemia, and opportunistic infections of the intestinal tract [8]. That is, the cause of death of Bmi-1^-/-^ mice may be aspiration pneumonia caused by deterioration of oral hygiene in addition to deteriorated immune function due to hematopoietic defects.

## Study strength and limitations

The strength of this study is that by using SMME, a method developed for analyzing difficult-to-handle mucins, mucins were separated and the detailed glycan structure that modifies mucins was clarified. Further, the combination of this approach with studies on knockout mice lacking expression of Bmi-1 protein, a component of PRC1, revealed a part of the mechanism of mucin synthesis.

This study has two limitations involving the evaluation of SMG mucin in mice. First, the sample size was small. If the sample size was slightly larger, it might be concluded that the expression level of *Smgc* was significantly different between WT and Bmi-1^-/-^ mice. Second, it is not possible to comment from which mucin the major glycan structure of the SMG mucin in Bmi-1^-/-^ mice is derived. The expression level of *Smgc* was significantly increased in Bmi-1^-/-^ mice but the mucin bands detected in the AB-stained SMME membrane could not be identified as Smgc. Since these mucin bands reacted with the anti-Muc10 antibody, it is speculated that the major glycan structure of Bmi-1^-/-^ mice may be derived from Muc10 or both Muc10 and Smgc.

## Conclusions

Using SMME to analyze mucins revealed that the polycomb protein Bmi-1 regulated mucin levels in the SMG by suppressing the expression of the mucin gene and that Bmi-1 also regulated mucin *O*-glycosylation via suppression of the glycosyltransferase gene in the SMG and was able to clarify in part the mechanism of mucin synthesis. In the future, it is expected that further elucidating the details of glycan structures in mucin that bind to adhesion proteins of various microorganisms will be a stepping stone for explaining the functions of mucin in maintaining oral hygiene. These results expand our current understanding of the molecular mechanisms underlying oral hygiene maintenance and open up new possibilities for the prevention and treatment of various oral diseases, including aspiration pneumonia, which is a leading cause of death in the elderly.

## Supporting information

S1 Fig SMME and MALDI MS analysis of SMG mucin in WT and Bmi-1^-/-^ mice. (A) (a) Comparison of SMG mucins of WT mice and Bmi-1^-/-^ mice (9 weeks) separated by SMME. All bands are from different individuals. (b) Comparison of SMG mucins of WT and Bmi-1^-/-^ mice (4–6 weeks) separated by SMME. All bands are from different individuals. Reference mucin: porcine gastric mucin (PGM). (B) MALDI MS analysis of the bands from WT and Bmi-1^-/-^ mice. The numbers indicate peaks permethylated glycans numbered in Table 1.
(EPS)

S2 Fig Antibody staining of SMME membranes from SMG fractions of WT and Bmi-1^-/-^ mice. (a) SMME membranes were blotted with antibodies against Mucin 1, Muc 10/Prol 1, Muc 19, and Smgc. (b) SMME membrane was blotted with antibodies against Muc 10/Prol 1. The SMG samples in (a) and (b) correspond to (a) and (b) in S1A Fig, respectively. The SMME experimental conditions are the same as in S1A Fig.
(EPS)

S3 Fig Validation of the specificity of the anti-*Smgc* antibody. (a) SMGs from 3-day old WT mice were used as positive controls for *Smgc* expression (red) via immunohistochemical analysis. DNA was stained with DAPI (blue). Red blood cells were stained non-specifically positive. Scale bar, 20 μm. (b) Pancreases from 3-day old WT mice were used as negative controls for *Smgc* expression (red) via immunohistochemical analysis. DNA was stained with DAPI (blue). Scale bar, 20 μm.
(EPS)

S1 Raw images (PDF)
