# Supplementary figures and images for: Bmi-1 regulates mucin levels and mucin O-glycosylation in the submandibular gland of mice
Source: PLoS One. 2021 Jan 19;16(1):e0245607. doi: 10.1371/journal.pone.0245607 (PMC7815129; doi:10.1371/journal.pone.0245607)

Fig 2

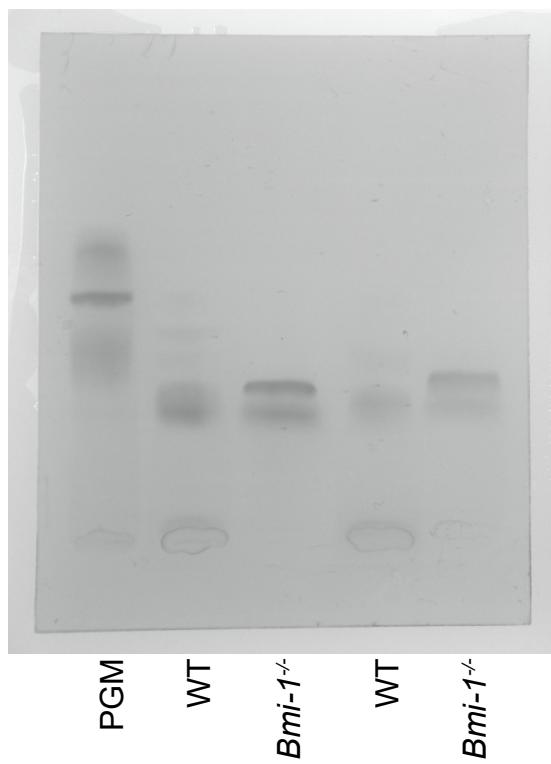

S1 Fig A (a)

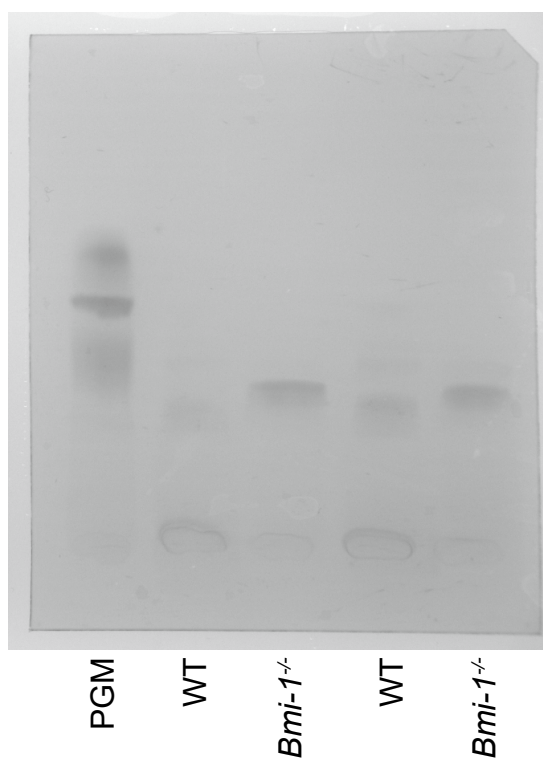

S1 Fig A (b)

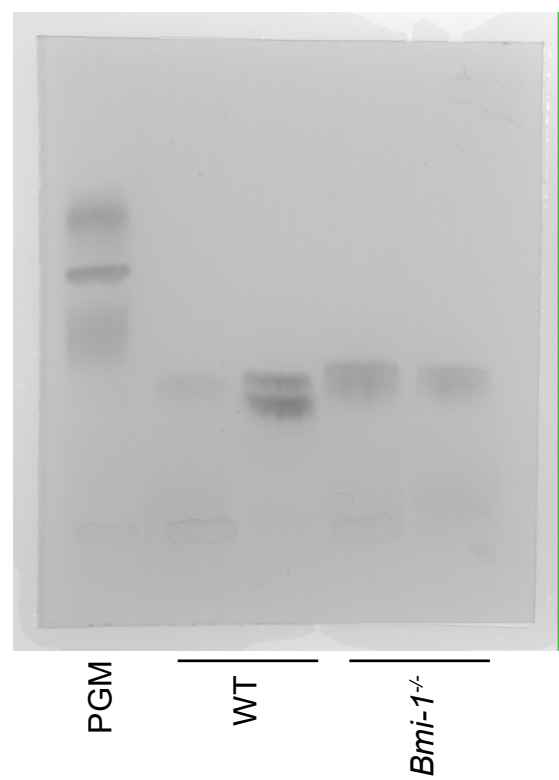

Fig 3C

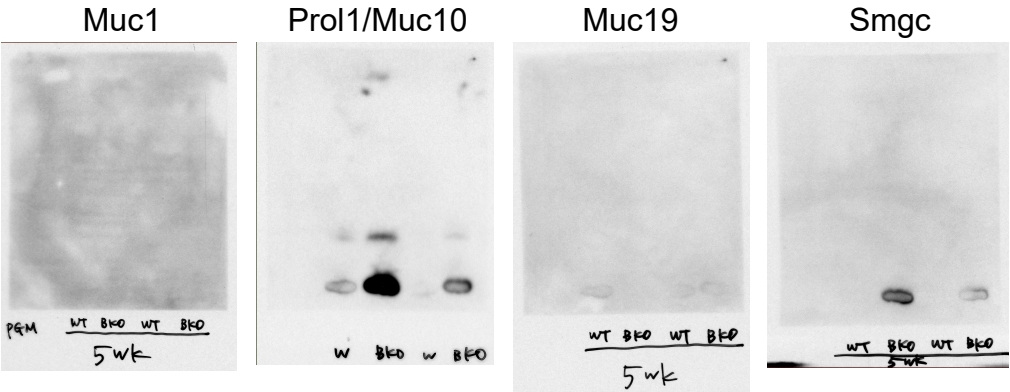

S2 Fig (a)

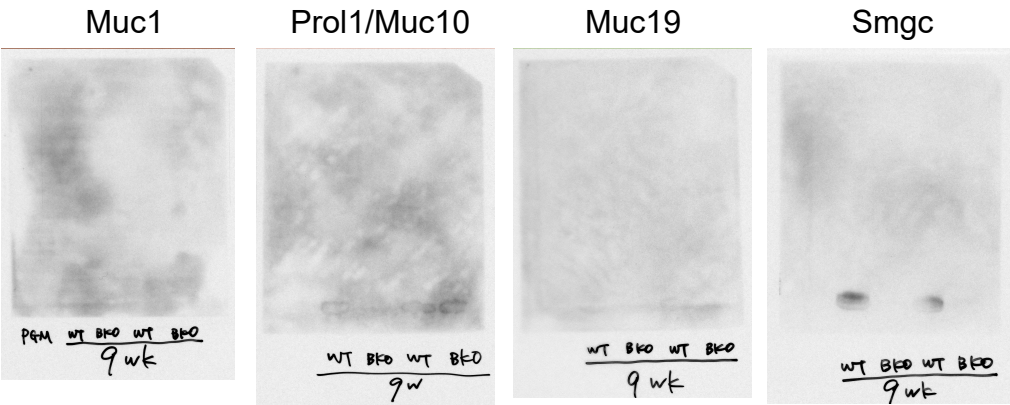

S2 Fig (b)

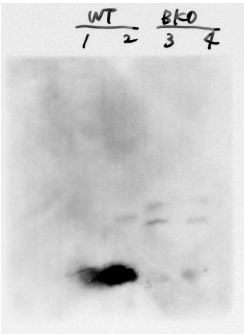

Supplement: S1 Raw images — (PDF) [file pone.0245607.s004.pdf]
